# Severe Leptospirosis Similar to Pandemic (H1N1) 2009, Florida and Missouri, USA

**DOI:** 10.3201/eid1706.100980

**Published:** 2011-06

**Authors:** Yi-Chun Lo, Kristina W. Kintziger, Henry J. Carson, Sarah L. Patrick, George Turabelidze, Danielle Stanek, Carina Blackmore, Daniel Lingamfelter, Mary H. Dudley, Sean V. Shadomy, Wun-Ju Shieh, Clifton P. Drew, Brigid C. Batten, Sherif R. Zaki

**Affiliations:** Author affiliations: Centers for Disease Control and Prevention, Atlanta, Georgia, USA (Y.-C. Lo, S.V. Shadomy, W.-J. Shieh, C.P. Drew, B.C. Batten, S.R. Zaki);; Missouri Department of Health and Senior Services, Jefferson City, Missouri, USA (Y.-C. Lo, S.L. Patrick, G. Turabelidze);; Florida Department of Health, Tallahassee, Florida, USA (K.W. Kintziger, D. Stanek, C. Blackmore);; Jackson County Medical Examiner’s Office, Kansas City, Missouri, USA (H.J. Carson, D. Lingamfelter, M.H. Dudley)

**Keywords:** leptospirosis, influenza A virus, subtype H1N1, Florida, Missouri, letter

**To the Editor:** Leptospirosis is caused by pathogenic spirochetes of the genus *Leptospira* and transmitted through direct contact of skin or mucous membranes with urine or tissues of *Leptospira*-infected animals or through indirect contact with contaminated freshwater or soil. Leptospirosis shares common clinical signs with influenza, including fever, headache, myalgia, and sometimes cough and gastrointestinal symptoms. During 2009, acute complicated influenza-like illness (ILI) and rapid progressive pneumonia were often attributed to pandemic (H1N1) 2009; however, alternative final diagnoses were reported to be common (*1*). We report 3 cases of severe leptospirosis in Florida and Missouri with clinical signs similar to those of pandemic (H1N1) 2009.

Patient 1 was a 40-year-old Florida man who sought treatment at an emergency department after a 4-day history of fever, myalgia, calf pain, malaise, and headache in July 2009. ILI was diagnosed. Laboratory testing was not performed, and the patient was instructed to take ibuprofen. Three days later, jaundice developed. He was admitted to an intensive-care unit with a diagnosis of hepatitis and acute renal failure. The man raised horses, goats, and chickens on his farm and was frequently employed to control rat infestations at an auto parts store and warehouse. Leptospirosis was suspected. Doxycycline was administered, and the man recovered and was discharged on the eighth day of hospitalization. *Leptospira*-specific immunoglobulin M antibodies were detected by dot blot (ARUP Laboratories, Salt Lake City, Utah, USA) on the second of paired consecutive blood specimens.

Patient 2 was a 17-year-old Missouri woman with a history of obesity. She was hospitalized in August 2009 with a 5-day history of fever, myalgia, calf pain, malaise, headache, nausea, vomiting, dyspnea, and cough, complicated by acute renal failure. The diagnosis on admission was viral infection. On the third day of hospitalization, severe pneumonia and respiratory failure developed, and she was administered vancomycin, piperacillin/tazobactam, levofloxacin, and doxycycline. She died the same day. Ten days before illness onset, she had swum in a creek near her residence.

Patient 3 was a 59-year-old Florida man with a history of obesity and diabetes mellitus. He sought treatment at a clinic in September 2009 and reported a 5-day history of fever, myalgia, malaise, nausea, abdominal pain, and dyspnea. He was treated for gastritis. Two days later, he came to an emergency department and was admitted to the hospital with severe pneumonia and multiorgan failure; he died the next day. The man had frequently engaged in activities to control rat infestations on the farm where he raised chickens, pigs, and goats.

Although patients 2 and 3 were neither tested nor treated for influenza before they died, their clinical signs and rapidity of death prompted postmortem suspicion of pandemic (H1N1) 2009. Autopsies were performed and formalin-fixed tissues were submitted to the Centers for Disease Control and Prevention (Atlanta, GA, USA). Histopathologic evaluation of both patients demonstrated extensive pulmonary hemorrhage and interstitial nephritis (Figure, panels A and B), features consistent with leptospirosis. Immunohistochemical tests for leptospirosis, spotted fever group rickettsiae, and influenza A were performed on multiple tissues obtained from patients 2 and 3. Immunohistochemical evidence of leptospiral infection was identified in lung, liver, kidney, heart, and spleen tissue in both patients (Figure, panels C and D).

**Figure:**
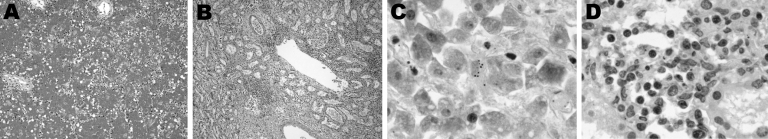
Photomicrographs of lung, liver, and kidney sections from patient 2 during study, Missouri and Florida, USA, 2009. Hematoxylin and eosin stain showed pulmonary hemorrhage (A) (original magnification ×10) and interstitial nephritis (B) (original magnification ×5), 2 characteristic pathologic findings of leptospirosis. Immunohistochemical testing showed scattered granular leptospiral antigens in liver (C) and kidney (D) (original magnification ×63).

These cases of severe leptospirosis were reported during the 2009 influenza pandemic. Although pulmonary hemorrhage (experienced by patients 2 and 3) is increasingly recognized as a severe manifestation of leptospirosis (*2*), it is also a known complication of influenza (*3*). ILI was initially diagnosed in patient 1, but symptom progression and clinical complications, combined with a history of animal exposure, prompted the physician to consider leptospirosis and to initiate appropriate antimicrobial drug therapy.

Autopsies are critical in determining the reasons for death after undiagnosed illness. Pulmonary involvement in cases of leptospirosis is characterized by congestion and hemorrhage, usually without prominent inflammatory infiltrates (*4*); pulmonary involvement in cases of severe pandemic (H1N1) 2009 typically manifests as diffuse alveolar damage (*5*). Postmortem diagnosis of leptospirosis was supported by characteristic histopathologic findings, including pulmonary hemorrhage and interstitial nephritis, and was confirmed by immunohistochemical tests. Our report illustrates the need for autopsies in unexpected deaths, even if the cause appears obvious in a specific clinical and epidemic setting.

Leptospirosis ceased being nationally notifiable in the United States in 1994 and is likely underdiagnosed because it is not routinely considered in differential diagnoses. However, outbreaks with exposures similar to the case-patients we studied have been periodically reported in the United States (*6**–**8*). Because leptospirosis commonly manifests as acute febrile illness, cases can be underrecognized during infectious-disease epidemics (e.g., dengue) (*9*). Leptospirosis should be included in the differential diagnosis of acute febrile illness in the United States and other industrialized countries. Epidemiologic clues include recreational or occupational water exposure; animal exposure (including rodents) in the home or the workplace, travel to tropical areas, and water exposure during travel. These risk factors for leptospirosis are increasing in industrialized countries (*10*). Thorough patient-history reviews and consideration of alternative diagnoses are needed for cases of respiratory illness during an influenza pandemic.
